# Fate of uptaken host proteins in *Taenia solium* and *Taenia crassiceps* cysticerci

**DOI:** 10.1042/BSR20180636

**Published:** 2018-07-06

**Authors:** Jeanette Flores-Bautista, José Navarrete-Perea, Gladis Fragoso, Ana Flisser, Xavier Soberón, Juan P. Laclette

**Affiliations:** 1 Department of Immunology, Institute for Biomedical Research, Universidad Nacional Autónoma de México, Ciudad de México, México; 2 Department of Parasitology, School of Medicine, Universidad Nacional Autónoma de México, Ciudad de México, México; 3 Instituto Nacional de Medicina Genómica, Ciudad de México, México

**Keywords:** calcareous corpuscles, host proteins, Mass spectrometry, metabolically labeled proteins, Taenia crassiceps, Taenia solium

## Abstract

During the study of host–parasite relationships in taeniid parasite diseases, including cysticercosis and hydatidosis, reports have described the presence of host proteins in the cyst fluid and tissue of metacestodes. However, the fate or role of host elements inside the parasite remains barely explored. After the publication of genomes of four cestode species, it became clear that these organisms possess a limited biosynthetic capability. The initial goal of the present study was to determine if uptaken host proteins could be a source of essential amino acids for cysticerci. To track the utilization of uptaken proteins, we added metabolically labeled IgG-^3^H and GFP-^3^H to the culture medium of *Taenia crassiceps* cysticerci. Incorporation of labeled amino acid was evaluated by fluorography in cysticerci extracts. Our results showed that the use of uptaken proteins by cysticerci as a source of amino acids appeared negligible. Exploring alternative fates for the host proteins, proteomic analysis of the protein matrix in calcareous corpuscles was carried out. Since *T. crassiceps* does not contain calcareous corpuscles, proteomic analyses were performed in corpuscles of *Taenia solium* cysticerci. Our results demonstrated that host proteins represented approximately 70% of protein content in the calcareous corpuscles. The presence of the two major uptaken host proteins, namely albumin and IgG, was also demonstrated by Western blot in the matrix of corpuscles. Our findings strongly suggested that the uptake and disposal of host proteins involve calcareous corpuscles, expanding the physiological role of these mineral concretions to a far more important level than previously proposed.

## Introduction

During the last decades, considerable advances for understanding host–parasite relationship in taeniid helminths have been achieved using a murine model of cysticercosis based on *Taenia crassiceps* [1]. This parasite has the advantage of its facility for maintenance under laboratory conditions, through intraperitoneal passage of cysticerci from infected to healthy mice [2,3]. The analysis of four tapeworm genomes has revealed highly simplified and host-dependent organisms [4]. Taeniids show a very limited biosynthetic metabolism, acquiring sugars, most amino acids (L, K, H, I, M, F, T, W, V, S, and P), nucleosides and fatty acids from the host, to produce its own macromolecules [4]. In contrast, these taeniids have great capability to uptake nutrients; cysticerci absorb and consume large quantities of glucose through transporters TGTP1 and TGTP2 and store the excess as glycogen [5]. A similar phenomenon occurs with the acquisition of fatty acids and cholesterol from the host environment [6,7]. Amino acid absorption in *T. crassiceps* was reported several decades ago, through the proposal of three mechanisms specific for neutral, basic, and acidic amino acids [8,9]. Analysis of the taeniid genomes also revealed the presence of coding genes for amino acid transporters [4].

Cysticerci are larval forms possessing a fluid-filled vesicle; the presence of host proteins in the vesicular fluid (VF) of cysticerci is a well-known fact [10–15]. We have also reported that host proteins might represent 11–13% of the protein content in the vesicular fluid of swine cysticerci, with albumin and immunoglobulins being the most abundant proteins [16]. More recently, using high-throughput proteomics, we identified 891 proteins of host origin from a total of 4259 that were identified and quantified in a *Taenia solium* cysticerci whole protein extract [17]; thus, host proteins represent 20% of total parasite protein species. The biological role and fate of the uptaken host proteins have barely been studied. Uptake of host albumin has been proposed to be involved in the maintenance of osmotic pressure [14]. In the case of uptaken haptoglobin and hemoglobin, the parasite appears to take advantage of their normal function in the host for its own benefit, we have proposed that these and other host iron chaperons are used by cysticerci to fulfill its iron requirements [18]. Uptake of immunoglobulin has been proposed as a mechanism of immune evasion and even as a source of amino acids [19].

Since host proteins are abundant in cysticerci tissues, the aim of the present study was to elucidate their fate in the larval tissue, using *T. solium* and *T. crassiceps* cysticerci. Initially, we evaluated if uptaken metabolically radiolabeled host immunoglobulin G (IgG-leucine^3^H) acted as a source of essential amino acids in *T. crassiceps* cysticerci. For this, we tracked the incorporation of one essential amino acid (leucine-^3^H) as a building block for the synthesis of cysticerci own proteins. For comparison, we used another metabolically radiolabeled protein that is not related to the parasite, the green fluorescent protein (GFP-leucine-^3^H). Our results showed that the use of uptaken proteins as a source of amino acids was remarkably low by cysticerci. Searching for an alternative fate for host proteins, we carried out proteomic analyzes of calcareous corpuscles (CC) in *T. solium*. CC are widely distributed in larval and adult forms of cestodes; these structures are mineral deposits of phosphate salts onto an organic matrix of proteins, polysaccharides and lipids, involved in gathering toxic metabolites and other materials into an inert structure [20,21]. Our results showed that host proteins constitute the main component, up to 70%, of the protein matrix. The presence of the two major uptaken host proteins, namely albumin and IgG, in the matrix of the corpuscles was also demonstrated by Western blot. Thus, a major fate for the uptaken host proteins seems to be its coalescence within these mineral concretions. Our results expand the physiological role of calcareous corpuscles and raise questions as to the design of cysticerci as biological devices.

## Materials and methods

### Biological specimens

For the *in vitro* protein uptake assays, *T. crassiceps* (ORF strain) cysticerci maintained through intraperitoneal passage in female mice BALB/cAnN strain were used [1]. Cysticerci were collected from the peritoneal cavity of infected mice after humanitarian killing and washed several times with sterile phosphate buffered saline, pH 7.4 (PBS) before *in vitro* culture. All procedures involving mice were carried out in accordance to the institutional guidelines for the care and use of laboratory animals (CICUAL permit No. ID199).

For the isolation of calcareous corpuscles, *T. solium* cysticerci were dissected from skeletal muscle of naturally infected pigs, after humanitarian killing, in accordance with institutional guidelines from the School of Veterinary Medicine and Zootechnia, UNAM. The inflammatory capsules surrounding cysticerci were removed and the parasites were washed several times with ice-cold PBS and frozen (without PBS) at −70°C until use.

### Metabolic labeling of mouse IgG and recombinant GFP

A murine hybridoma producing monoclonal IgG antibodies (against an unrelated antigen) was grown in RPMI-1640 medium (Biowest) supplemented with 10% fetal bovine serum (FBS). A new culture of this hybridoma was initiated at a density of 1 × 10^5^ cells/ml in a medium added with leucine-^3^H (Leu-^3^H) at 5 µCi/ml (Perkin-Elmer) and maintained for 14 days. After centrifugation of hybridoma cells, the supernatant was collected and mixed 1:1 (v/v) with sodium phosphate buffer (20 mM), pH 7.4. Isolation of the metabolically labeled mouse IgG (IgG-^3^H) was carried out by affinity chromatography through a column of protein G coupled to Sepharose 4B (Sigma-Aldrich Co). Western blot assays using crude extracts of *T. crassiceps* and *T. solium* cysticerci reacted against the supernatant of the hybridoma showed no recognition of any parasite’s protein (not shown).

Metabolically labeled green fluorescent protein (GFP-^3^H) was produced by growing a recombinant strain of *Escherichia coli* [22]. Bacteria were grown in Luria-Bertani medium containing 5 µCi/ml of Leu-^3^H (Perkin-Elmer) for 24 h. Bacteria were harvested and suspended in lysis buffer of guanidine 5 M, Tris 10 mM pH 7.4 and complete protease inhibitors cocktail (Roche), sonicated 3 min (six passes of 30 s) within an ice bath and centrifuged for 30 min at 4000 ***g***. Isolation of the GFP was carried out by affinity to nickel, through a HiTrap IMAC FF column (GE Healthcare), according to the manufacturer’s instructions.

The specific activity of the IgG-^3^H and GFP-^3^H was determined by dissolving aliquots of each one in Tritosol [23] and evaluating the amount of radioactivity in a liquid scintillation counter (Beckman Coulter LS6500). Radiolabeled proteins were also evaluated by fluorography [24]. Briefly, 10 µg of extracts were resolved by SDS-PAGE, the gels were soaked twice for 30 min with dimethyl sulfoxide (DMSO) and then in 20% (w/w) PPO (2,5-diphenyloxazole) for 3 h. Finally, DMSO was removed with water and the gel was dried and exposed to an X-ray film at −70°C for about 10 days before development.

### *In vitro* culture of *T. crassiceps* cysticerci in the presence of IgG-^3^H and GFP-^3^H

Groups of 20 cysticerci were precultured for 3 days in ten volumes of a minimal medium for cysticerci (MMC) (Supplementary File S1) containing the salt base of RPMI-1640 without amino acids. Preculture medium was replaced every 24 h. For the evaluation of the uptake and use of metabolically labeled proteins, cysticerci were cultured during nine more days in 1 ml of MMC supplemented with 2% albumin and 500 µg of IgG-^3^H (430,125 CPM) or GFP-^3^H (9225 CPM) at 37°C with an atmosphere of 5% CO_2._ In order to evaluate the biosynthetic activity of the parasites under culture conditions used in these experiments, another group of cysticerci, from the same stock, was cultured in identical medium added only with 30 µCi of Leu-^3^H.

At the end of each culture, cysticerci were collected and washed as above. The VF was obtained by cutting the bladder wall with a scalpel on a Petri dish and mixed 1:1 (v/v) with lysis buffer (7 M urea, 2 M thiourea, 4% CHAPS, 10 mM Tris) and protease inhibitors cocktail. A tissue extract (TE) was obtained by homogenization of cysticerci in a lysis buffer using a Benchmark D1000 homogenizer. The homogenate was centrifuged for 15 min at 14000 ***g*** and the supernatant collected. The TE, VF, and culture medium (CM) collected were frozen at −70°C until use. To ascertain if radioactivity had been incorporated into newly synthesized cysticerci proteins, 100 µg of each extract were resolved by SDS-PAGE using 10% polyacrylamide gels and processed for fluorography as described above, exposing the dried gels for approximately 30 days.

### Functional activity of the uptaken mouse IgG

A crude extract was obtained through homogenization of cysticerci in PBS (1:1, v/v) adding the protease inhibitor cocktail. Extracts were centrifuged 15 min at 14000 ***g*** and the supernatant collected. The total extract was applied to a Protein G-Sepharose 4B column. Bound IgG was eluted using 0.1 M glycine, pH 2.3, and immediately neutralized with Tris 1 M, pH 11. Fractions containing the bound IgG were concentrated using an Amicon system (10 kDa cutoff) and washed several times using PBS, pH 7.4. The purified IgG was quantified by the noninterfering protein assay kit (GBiosciences) and tested for antibody activity against TE and VF by Western blot using rabbit anti-mouse IgG coupled to peroxidase (Sigma-Aldrich) as secondary antibody. The antigen–antibody reaction was developed using a West-Femto chemiluminiscence kit (Thermo Scientific) following manufacturer’s instructions.

### Calcareous corpuscles protein extracts from *T. solium* cysticerci

The CC were isolated after homogenization of *T. solium* cysticerci in PBS as above. This species of parasite allows isolation of enough corpuscles for proteomic analysis. Two stocks of cysticerci obtained from naturally infected pigs were used; the homogenates were centrifuged at 14000 ***g*** for 30 min at 4°C to sediment CC in the bottom of the tube. Cell debris was removed by resuspending pellets in ultrapure water (Milli-Q, Millipore Co) followed by centrifugation; this rinsing procedure was repeated at least eight times until clean whitish pellets of CC were obtained. The two pellets were resuspended separately in 0.5 ml of 0.1 N HCl to remove proteins that are not part of the matrix but adsorbed onto the CC surface during cysticerci homogenization. Finally, CC were centrifuged and then dissolved in 2 ml of 0.1 N HCl. Dissolved CC material was concentrated using ultrafiltration Amicon vials (3 KDa cutoff) and resuspended in 150 µl of buffer containing 7 M urea, 2 M thiourea, 4% CHAPS, and 10 mM Tris. Protein content was quantified by noninterfering protein assay (GBiosciences).

### Western blot of calcareous corpuscles protein extracts

Samples of 15 µg of each *T. solium* cysticerci CC protein extract were resolved in 15% SDS-PAGE and transferred onto a nitrocellulose membrane. In the case of porcine serum, 5 μg were applied to the lane. Membranes were blocked using 10% skim milk in PBS and reacted with sheep α-albumin (Abcam ab186525) and then with a rabbit α-sheep-IgG (Abcam ab6747), or with a rabbit polyclonal α-mouse IgG serum (A9044 Sigma-Aldrich).

### Mass spectrometry of the calcareous corpuscles protein extracts

Prior to analysis, CC protein extracts were reduced using 10 mM dithiothreitol (DTT, BioRad) for 1 h and alkylated with 15 mM iodoacetamide (BioRad) for 30 min. Iodoacetamide was quenched using 15 mM DTT. Each sample was digested with modified porcine trypsin and prepared for LC/MS/MS. Samples were injected into an Acquity UPLC-BEH-C18 column (Waters, Milford, MA), which was coupled in-line with a LTQ Orbitrap XL (Thermo Fisher Scientific, Waltham, MA) equipped with an electrospray ion source. The samples were equilibrated in solvent A containing 100% H_2_O and 0.1% formic acid and eluted through a gradient with solvent B: 100% acetonitrile and 0.1% formic acid (see below). A sample of 3 µl of trypsin digested proteins was trapped using pre-column (Symmetry® C18, 5 µm, 180 µm × 20 mm, Waters), which was then switched in-line onto a 10-cm capillary UPLC column (100 um ID BEH-C18 1.7 µm particle size). The column was enclosed in a column heater operating at 35°C. After loading, peptides were separated with a 60-min gradient at a flow rate of 400 nl/min. The gradient was as follows: 3–50% Solvent B, 30 min; 50–85%, 1 min; 85%, 7 min and 3%, 22 min. Eluted peptides were directly electrosprayed into the mass spectrometer through a standard coated silica tip (NewObjective, Woburn, MA).

The LTQ was operated in data-dependent acquisition mode to automatically alternate between a full scan in a range of *m/z* 400–2000, and the three most intense ions were sequentially isolated and fragmented using collision-induced dissociation (CID). CID was performed with helium as collision gas at normalized collision energy of 40% and 10 ms of activation time. Data acquisition was controlled by Xcalibur 2.0.7 software (Thermo Fisher Scientific). Tandem mass spectra, obtained from the LTQ Orbitrap XL, were extracted by Proteome Discoverer version 1.3 (Thermo Scientific) and obtained peptide sequences were searched for Sequest against *T. solium* and *Sus scrofa* genome databases to identify the proteins of origin.

All files generated by Sequest were searched with the following parameters: Peptide tolerance was allowed to be 1.6 Da, fragment mass tolerance was ±0.8 Da, and a maximum two missed cleavages allowed. Carbamidomethylation of cysteine was set as static modification and methionine oxidation was set as variable modification.

### MS data analysis

Relative protein abundance, represented by normalized spectral abundance factors, was calculated by the number of spectral counts (SpC) identifying a protein, divided by the protein’s length, divided by the sum of SpC for all proteins in the experiment [25]. Gene ontology (GO) analysis for the host (*Sus scrofa*) was performed using the PantherGO algorithm [26]. Taenia proteins were annotated and analyzed for GO with Blast2GO [27].

## Results

### Uptake and use of metabolically labeled IgG and GFP by *T. crassiceps* cysticerci maintained under *in vitro* culture

**Figure 1:**
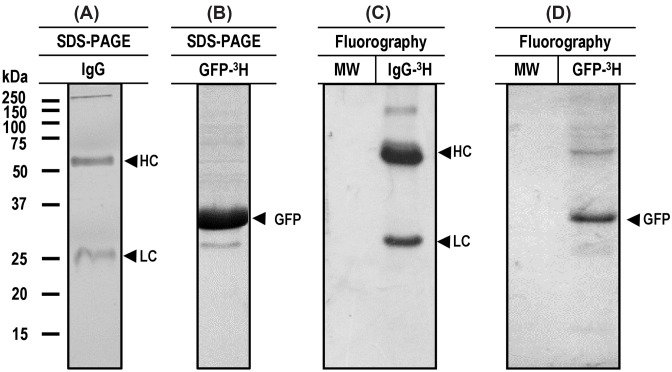
Metabolically labeling and purification of mouse IgG and GFP After separate *in vitro* cultures of mouse hybridoma and recombinant bacterial cells, the metabolically labeled IgG and GFP were purified by affinity chromatography using columns with protein G and Nickel Sepharose 4B respectively. The purity of IgG (**A**) and GFP (**B**) was evaluated by SDS-PAGE. The radioactive labeling was evaluated by fluorography on SDS-PAGE treated gels. In both cases, radioactive labeling of IgG heavy and light chains (**C**) and GFP (**D**) was detected in the expected molecular sizes; MW, molecular weight marker.

For the protein uptake assays, *T. crassiceps* cysticerci were used to take advantage of their ready availability. IgG-^3^H and GFP-^3^H were produced separately under *in vitro* conditions. Purity of the two metabolically labeled proteins resulted > 81% as estimated by densitometry of Coomassie Blue stained gels (Figure 1); IgG-^3^H was observed as the two-characteristic heavy (50 kDa) and light (25 kDa) chains (Figure 1A), an additional band was observed at 150 kDa, which apparently corresponded to the whole immunoglobulin. GFP-^3^H was also observed at the expected (27 kDa) size with additional bands of lower abundance (Figure 1B). Tritium labeling of the two proteins was detected by fluorography of the dried gels (Figure 1C,D).

**Figure 2:**
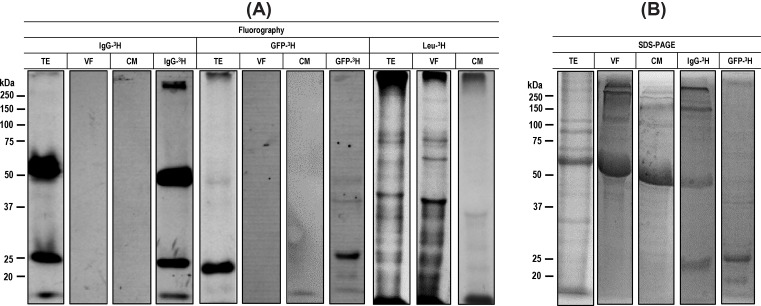
Electrophoresis and fluorography of protein extracts from *T. crassiceps* cysticerci maintained under *in vitro* culture in the presence of IgG-^3^H or GFP-^3^H Cysticerci were cultured in the presence of IgG-^3^H or GFP-^3^H and a paired group of cysticerci was cultured with Leu-^3^H for 9 days. After harvesting the cysts, samples of TE, VF, and CM were obtained. (**A**) All extracts were resolved in 10% SDS-PAGE and treated for fluorography. The X-ray films were exposed to dried gels for approximately 30 days. (**B**) SDS-PAGE patterns of cysticerci’s extracts stained with Coomassie blue.

To track the uptake of host proteins and their use as a source of amino acids by *T. crassiceps* cysticerci, IgG-^3^H or GFP-^3^H was added to the culture medium where cysticerci were maintained *in vitro* for 9 days. The TE, VF, and CM protein extracts were resolved by SDS-PAGE (Figure 2B). Radioactive label of the proteins in the extracts was determined by fluorography. In the case of cysticerci cultures in the presence of IgG-^3^H, the expected heavy and light chains were clearly observed at approximately 50 and 25 kDa in TE; the two bands were not detected in VF and CM (Figure 2A). Additional bands of lower size were barely observed underneath the 50 kDa band, suggesting a marginal degradation of the metabolically labeled IgG. Similarly, fluorography of cysticerci extracts cultured in the presence of GFP-^3^H showed the band corresponding to complete GFP (27 kDa) and several weak bands also observed in the purified GFP, also consistent with a limited proteolytic activity in cysticerci tissue. Again, the major 27 kDa band was not observed in VF or CM. In contrast with the lack of detection of IgG and GFP in the VF, SDS-PAGE of the same extracts showed the abundant presence of a 67 kDa protein in VF, consistent with BSA (Figure 2B), suggesting that this protein was uptaken from the culture medium, strongly supporting the concept that this protein acts as an osmotic regulator for cysticerci [14].

The *in vitro* maintenance during 9 days in a minimal culture medium posed the question as to the physiological state of cysticerci. To ascertain if the larvae remained capable for protein biosynthesis, a group of cysticerci was maintained *in vitro* in the same culture medium added with Leu-^3^H. The protein extracts obtained from this culture showed numerous radioactive bands ranging from 25 to more than 250 kDa, indicating that cysticerci retained a high biosynthetic activity under the culture conditions used in these experiments (Figure 2A).

### Functional assays of host IgG purified from *T. crassiceps* cysticerci’s crude extracts

**Figure 3:**
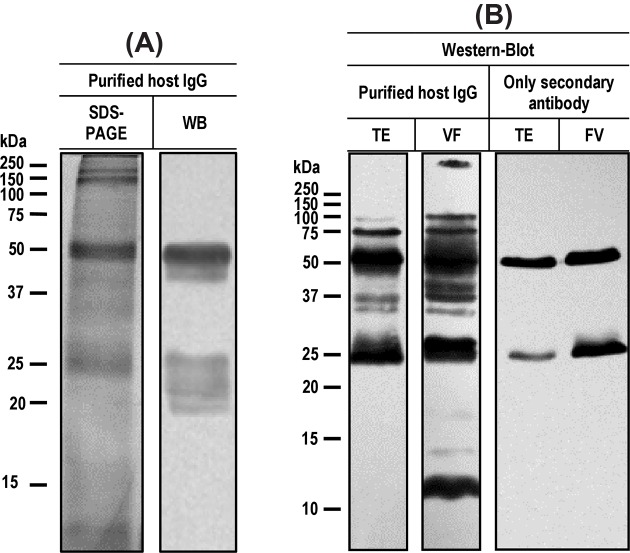
Purification and functional assays of the uptaken host IgG in protein extracts of *Taenia crassiceps* cysticerci (**A**) Purity of the bound IgG was evaluated by SDS-PAGE and Western blot using an anti-mouse IgG coupled to HRP. (**B**) Antibody function of the purified mouse IgG was evaluated through Western blot. Samples of *T. crassiceps* TE and VF were resolved through SDS-PAGE and then transferred to a nitrocellulose membrane. The IgG purified from cysticerci’s total protein extracts was used as primary antibody followed by a rabbit anti-mouse IgG coupled to HRP, as secondary antibody. The secondary antibody alone was also tested as control.

To evaluate if uptaken host immunoglobulins maintained an antigen binding activity, as recently described for *T. solium* [17], mice IgG was purified from a saline crude extract of *T. crassiceps* cysticerci (Figure 3). The purity of the isolated mouse IgG was evaluated by SDS-PAGE and Western blot, using rabbit anti-mouse IgG, the heavy and light chains of the IgG were readily detected (50 and 25 kDa) (Figure 3A). Antibody activity testing was also carried out by Western blot: tissue extracts and VF were resolved by SDS-PAGE and transferred onto a nitrocellulose membrane, then, membranes were incubated with IgG purified from *T. crassiceps* cyst; several bands were recognized (Figure 3B).

### Proteome profile of the *T. solium* calcareous corpuscles

The well-known abundance of immunoglobulins in cysticerci tissue and fluid contrasted with its marginal utilization as a source of amino acids observed above. An alternative fate for host proteins inside cysticerci was investigated. It is well known that CC are mineral concretions of phosphate salts deposited onto an organic matrix of proteins, polysaccharides, and lipids. Formation of CC has been proposed in taeniids as a mechanism of gathering toxic metabolites and other materials into an inert structure [20,21]. In spite of the fact that all above analyses were carried out using *T. crassiceps*, to facilitate the proteome analysis of CC we decided to use *T. solium* cysticerci that produce manageable amounts of CC. Our assumption was that the physiological role of these calcareous concretions, in closely related species of taeniid tapeworms, was alike. It is worth remembering that little is known about the protein composition of the CC organic matrix [28], therefore, we decided to analyze the protein composition of CC through mass spectrometry as well as through Western blotting using specific antibodies to host proteins (IgG and albumin).

Protein extracts of CC were obtained from two batches of *T. solium* cysticerci and then processed for mass spectrometry. From the peptide sequences obtained, a total of 636 and 760 proteins were identified in the two samples of CC, through search in the *T. solium* and *Sus scrofa* genome databases (Supplementary File S2). A remarkable finding was that approximately 70% of the proteins identified in both CC samples, 412 and 508, corresponded to host proteins, whereas only 224 and 252 proteins corresponded to *T. solium* in the first and second sample respectively. Variability of proteins identified in the two CC samples was considerably high, only 111 (73 host and 38 parasite) proteins were shared (Supplementary File S2); this means that 75–80% of the identified proteins were variable in the two samples of CC. However, GO analysis on the 636 and 760 host and parasite proteins identified in the two CC samples resulted in similar patterns of function (Supplementary Figure S1 and S2). For example, included in the 20 most abundant host and parasite proteins were those involved in transport mechanism in mitochondria (mitochondrial thiamine pyrophosphate carrier, S-adenosylmethionine mitochondrial carrier), transcription regulation (KRAB A domain containing protein, PC4 and SFRS1-interacting isoform X2, zinc finger 182 isoform X1), and regulation of cytoskeleton (twitchin, arfaptin-1 isoform X1, microtubule-associated 6 isoform X1, formin 2 isoform X7) (Supplementary File S3).

**Figure 4:**
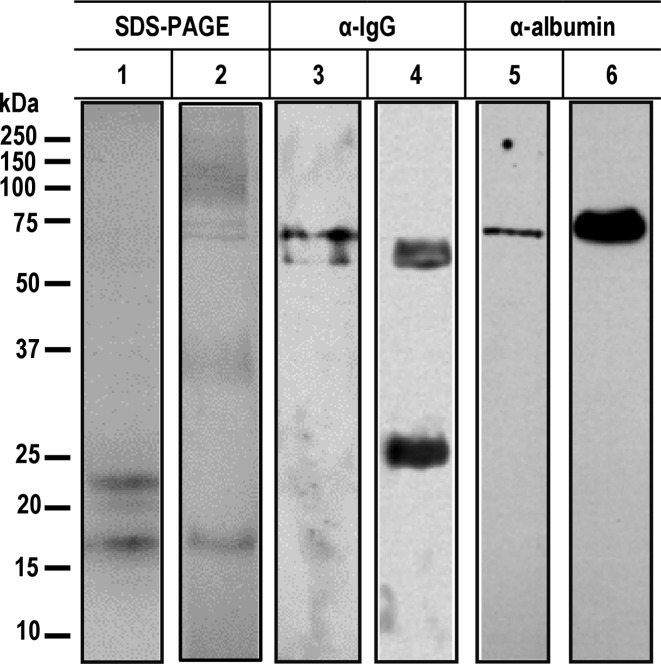
Immunological identification of host IgG and albumin in the protein matrix of calcareous corpuscles of *T. solium* cysticerci (1) Coomassie Blue staining of a protein extract of (15 μg) CC and (2) filver staining of porcine serum (5 μg). Western blot of protein extracts of CC (3 and 5) and porcine serum (4 and 6) reacted with polyclonal antisera directed against mouse IgG coupled to HRP (3 and 4) or a sheep α-albumin and then with a rabbit α-sheep-IgG coupled to HRP (5 and 6).

Another approach to the analysis of host proteins in the CC protein matrix was through Western blot using specific antibodies against porcine albumin and IgG (Figure 4). Results showed that both proteins were incorporated into CC protein matrix at least partially intact; albumin was detected in a 67 kDa band whereas IgG showed only a 50 kDa band corresponding to the heavy chain; the light chain was not detected.

## Discussion

The initial goal of the present study was to elucidate the fate of the abundant uptaken host proteins in taeniid larval tissue, particularly immunoglobulins, as a source of amino acids for newly synthesized cysticerci proteins or their disposal as waste in calcareous corpuscles. Several studies have reported the presence of host proteins inside the vesicular fluid and tissue of taeniid larval stages including *Echinococcus granulosus* [29–34], *T. crassiceps* [10,13,14,32], *T. taeniaformis* [10,12,32], *T. saginata* [11], and *T. solium* [16,17]. Surprisingly, the final use or fate of these host proteins remains poorly studied; albumin has been proposed to regulate the osmotic pressure in the vesicular fluid and/or scavenge reactive oxygen species in *T. crassiceps* [14], whereas hemoglobin, haptoglobin, and ferritin have been proposed to play a role for the parasite as iron chaperons, similar to the role they play in the host tissues [17,18]. In the present study, we cultured cysticerci in the presence of metabolically labeled IgG or GFP. Leu-^3^H allowed to track the incorporation of this amino acid residue onto cysticerci proteins because it is an abundant amino acid in all organisms and taeniid cysticerci are unable of synthetizing it [4]. Therefore, if the metabolically labeled proteins (IgG or GFP) were degraded up to free amino acids, the Leu-^3^H should be incorporated into *de novo* synthetized cysticerci proteins. A defined and minimal culture medium (MMC), without free amino acids, was specifically formulated here to favor the use of the residues released after degradation of the labeled protein. Cysticerci were precultured in the absence of free amino acids and proteins, in order to favor the release of host proteins in the freshly dissected cysticerci, as previously reported [14]; then, cysticerci were cultured in the same medium in the presence of one of the metabolically labeled proteins, only supplemented with albumin, to avoid cysticerci’s osmotic stress.

Unexpectedly, no new protein containing leucine-^3^H appeared to be synthesized out of the metabolically labeled IgG or GFP. If cysticerci incorporated Leu-^3^H from IgG or GFP into newly synthesized proteins, the amount was negligible and could not be detected by the methodology used in the present study. In fact, only a marginal part of IgG and GFP appeared degraded in our fluorography assays. It is worth mentioning that cysticerci maintained a high biosynthetic activity under the 9 days of *in vitro* culture used here, as shown in experiments where groups of cysticerci were maintained *in vitro* in the same culture medium added only with Leu-^3^H (Figure 2A).

Both findings suggest that digestion of IgG and GFP (and possibly other proteins), as a mechanism to obtain free amino acids for protein biosynthesis, seems to be negligible. Instead, cysticerci appeared to show a preference to absorb free amino acids from the environment; free amino acids are present in blood plasma and other body fluids at similar levels. High levels of free amino acids have been reported in hydatid fluid [35,36].

We reported long ago, the unspecific uptake of mouse immunoglobulin by *T. crassiceps* cysticerci [13]; more recently, we showed that host IgG uptaken by *T. solium* cysticerci maintain their antibody activity [17]. In this report, this finding for mouse IgG purified from *T. crassiceps* cysticerci was confirmed; several cysticerci’s antigens in the range of 12–250 kDa were clearly recognized by the affinity-purified uptaken IgG antibodies. In fact, several antigenic parasite bands were highly recognized by the purified host IgG in VF, suggesting that uptaken host antibodies play a role in diminishing the exposure of relevant parasite antigens.

Calcareous corpuscles are mineral concretions widely distributed in cestodes that might be involved in osmoregulation, neutralization of metabolic acidic products, or simply as reservoirs of inorganic material [37,38]. In the case of *T. solium*, CC are formed in the lumen of protonephridial ducts [20,21] and made up of mineral salts such as Ca, Mg, Mn and Cu, mainly calcium phosphates, deposited on a complex organic matrix composed of proteins, polysaccharides, and nuclei acids [39]. The composition of the organic matrix of CC has been poorly studied; an early report describes the presence of intact antigenic proteins as well as a calcium binding protein [28]. In this report, we explored the protein composition of CC through mass spectrometry. Over 600 proteins were identified in each sample. Surprisingly, approximately 70% of the identified proteins corresponded to host proteins. This finding raises the issue of the protein composition of the CC matrix to an entirely different level; perhaps CC can be conceived as a mechanism for the storage/disposal of the uptaken host proteins.

As an additional approach to explore the function of the host uptaken proteins, we carried out assays for the identification of the proteins in CC extracts through Western blot. Albumin and IgG were readily identified using specific antibodies; interestingly, both proteins appeared in the expected molecular size, indicating that both proteins remained intact in the CC matrix. This suggests that, at least, part of host proteins is enrooted toward CC without being degraded. The mechanism of this process remains entirely unknown.

We have described that uptaken host proteins might play a role for cysticerci, similar to the role they play in host tissues [16,17], or as described here, they might play a role as components of the protein matrix for the process of mineral deposition in CC. The present study, together with our previous findings of the notably high content of host protein species in the tissues and fluid of taeniid metacestodes, up to ∼21%, possibly align the uptake of host proteins with their disposal through CC [17].

Proteins that we identified in CC samples were extremely variable; cysticerci used for the isolation of CC came from different naturally infected pigs bred in small rural communities in remote areas of Mexico. Differences could be due to the fact that each sample was made up of many cysticerci from a single pig. To our knowledge, individual variability in the process of CC formation between individual cysticerci has not been explored. Other possibilities to explain the high variability in the protein composition of the CC includes factors such as time of infection, stage of the parasite, immune status of the host etc. [39,40].

Finally, we propose that the uptake, utilization, and disposal of host proteins clearly depict an unmatched host–parasite molecular intimacy in cysticercosis. Moreover, CC might play a physiological role far more important to the previously proposed. It appears that cysticerci uptake at least some intact host proteins and take advantage of their original function in the host tissues until they are simply caught up by salt deposition in the CC.

### Supporting information

**Table:** Minimal medium for cysticerci (MMC) composition

**Table:** 

**Table:** 

## Abbreviations

CC: calcareous corpuscles
CPM: counts per minute
CID: collision-induced dissociation
CM: culture medium
MMC: minimal medium for cysticerci
SpC: spectral counts
TE: tissue extract
VF: vesicular fluid
