# Supplementary material for: Fate of uptaken host proteins in Taenia solium and Taenia crassiceps cysticerci
Source: Biosci Rep. 2018 Jul 6;38(4):BSR20180636. doi: 10.1042/BSR20180636 (PMC6435539; doi:10.1042/BSR20180636)
Supplement: Supplementary file 1 [file bsr20180636_Supp1.pdf]

Minimal medium for cysticerci (MMC) composition

	g/L
Calcium Nitrate • $4H_2O$ (Sigma)	0.1
Magnesium Sulfate (anhydrous)	0.04884
Potassium Chloride	0.4
Sodium Bicarbonate	2
Sodium Chloride	6
Sodium Phosphate Dibasic (anhydrous)	0.8
D-Glucose	2
HEPES	5.96
Phenol Red • Na	0.0053
Proteins	
Bovine serum albumin (Sigma A3069)	20

Diamond Vitamin Tween® 80 Solution [1x]	
	mg/L
Inorganic salts	
Thiamine HCl	0.1375
Vitamins	
DL-α-Tocopherol	0.125
Biotin	0.1375
D-calcium pantothenate	0.1375
Choline chloride	6.9175
Cyanocobalamin	0.1
Ergocalciferol	1.375
Folic acid	0.1375
DL-α-Lipoic acid	0.1675
i-inositol	0.6925
Menadione	0.275
Niacin	0.265
Niacinamide	0.265
PABA	0.6925
Pyridoxal HCl	0.265
Pyridoxine HCl	0.265
Riboflavin	0.1375
Vitamin A acetate	1.375
Other	
Ethyl alcohol 190 proof	1.7515 ml

TweenTM 80	111.125

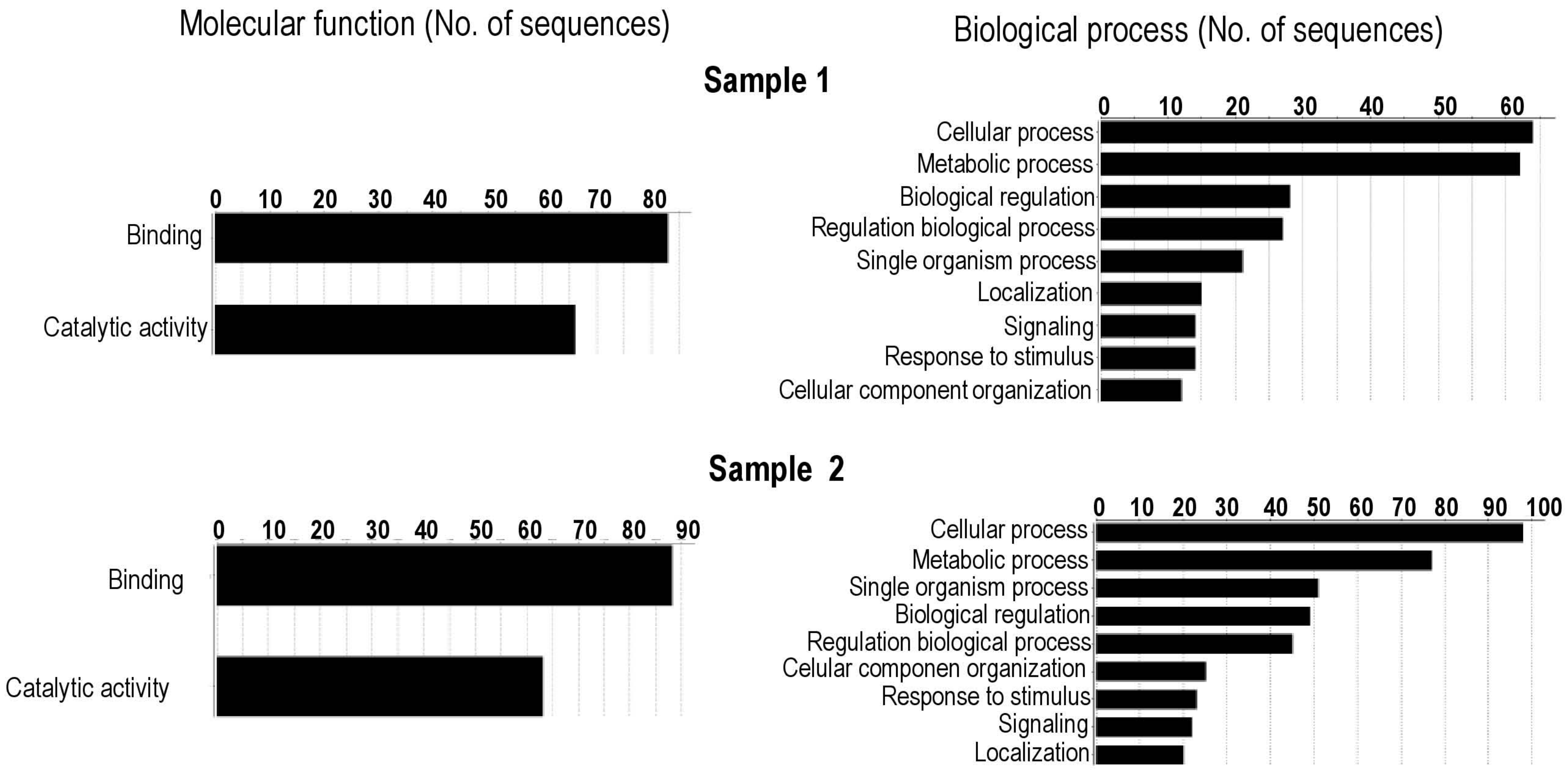
Molecular function (No. of sequences)
Biological process (No. of sequences)
Sample 1
0
10
20
30
40
50
60
70
80
Binding
Catalytic activity
0
10
20
30
40
50
60
Cellular process
Metabolic process
Biological regulation
Regulation biological process
Single organism process
Localization
Signaling
Response to stimulus
Cellular component organization
Sample 2
0
10
20
30
40
50
60
70
80
90
Binding
Catalytic activity
0
10
20
30
40
50
60
70
80
90
100
Cellular process
Metabolic process
Single organism process
Biological regulation
Regulation biological process
Celular componen organization
Response to stimulus
Signaling
Localization

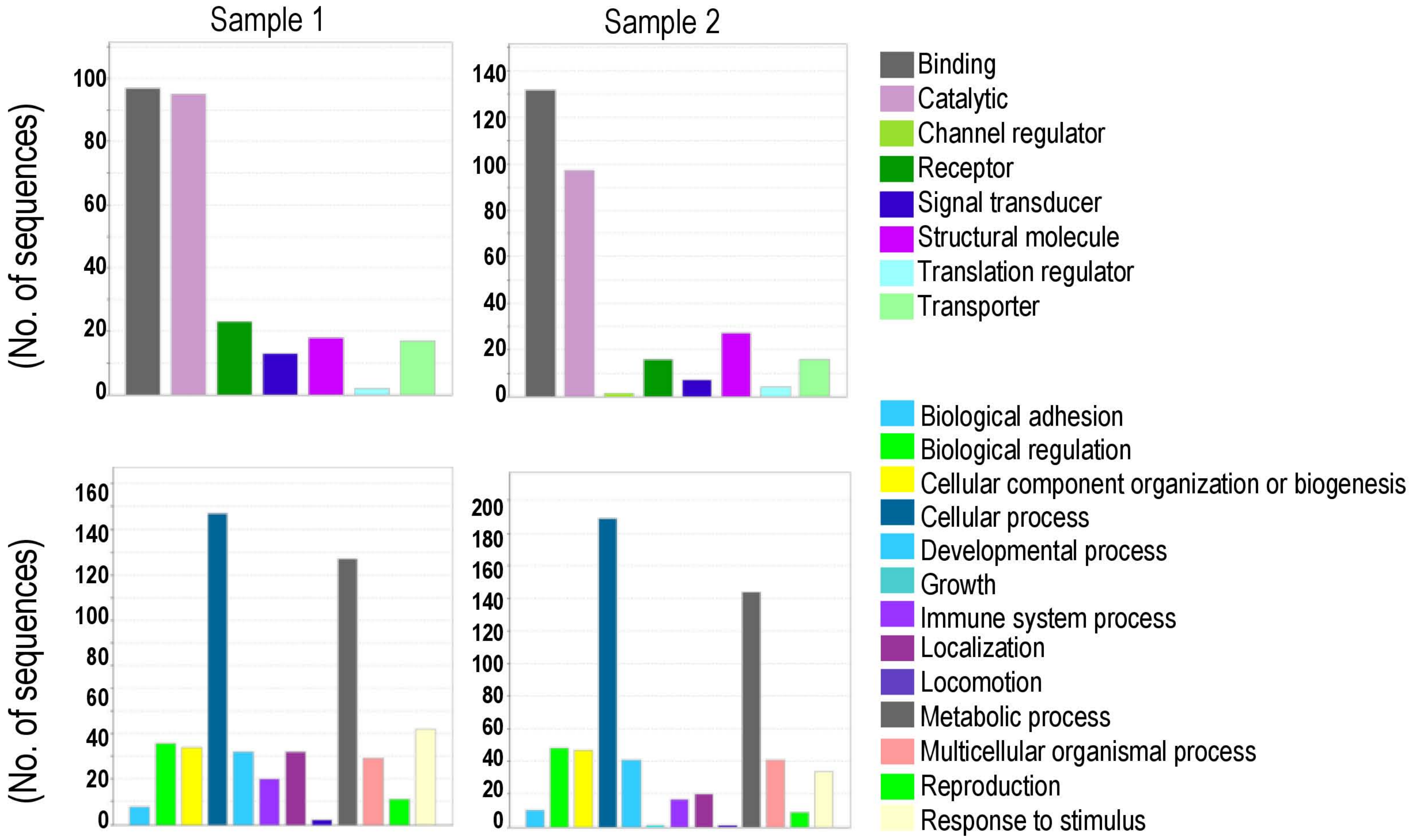
Sample 1
Sample 2
(No. of sequences)
(No. of sequences)
Binding
Catalytic
Channel regulator
Receptor
Signal transducer
Structural molecule
Translation regulator
Transporter
Biological adhesion
Biological regulation
Cellular component organization or biogenesis
Cellular process
Developmental process
Growth
Immune system process
Localization
Locomotion
Metabolic process
Multicellular organismal process
Reproduction
Response to stimulus
